# The Effect of Ginger Supplementation on the Improvement of Dyspeptic Symptoms in Patients With Functional Dyspepsia

**DOI:** 10.7759/cureus.46061

**Published:** 2023-09-27

**Authors:** Lemlem Gebremariam Aregawi, Mohammad Shokrolahi, Teferi Gebru Gebremeskel, Csiki Zoltan

**Affiliations:** 1 Institute of Nutrition, Faculty of Medical Sciences, University of Debrecen, Debrecen, HUN; 2 Public Health, College of Medicine and Health Sciences, Adigrat University, Adigrat, ETH; 3 Internal Medicine, University of Debrecen, Debrecen, HUN; 4 Flinders Health and Medical Research Institute (FHMRI), College of Medicine and Public Health, Flinders University, Adelaide, AUS

**Keywords:** functional disorders, dyspepsia, supplement, zingiber officinale, ginger

## Abstract

Background

Functional dyspepsia (FD) is a common gastrointestinal disorder with a higher occurrence in the Western world. Conventional medications are not effective for FD with patients turning to alternative herbal medicines such as ginger. This study aimed to evaluate the effect of ginger supplementation on the improvement of FD symptoms.

Methodology

A before-and-after clinical study was conducted at the University of Debrecen, Internal Medicine outpatient department over a period of four weeks. Two Swanson ginger supplements each at a dose of 540 mg/day before lunch and dinner were given to 51 FD patients. Wilcoxon signed-rank test was used to estimate the differences in FD symptoms after ginger supplementation and at the baseline. The p-value was determined to test the association between variables, with p-values <0.05 considered the cutoff for statistically significant association.

Results

In this study, after four weeks of ginger supplementation, we observed a significant change in most dyspepsia symptoms as follows: postprandial fullness (p = 0.033, 95% CI = 0.01-0.26), early satiety (p = 0.001, 95% CI = 0.10-0.37), epigastric pain (p = 0.000, 95% CI = 0.16-0.42), epigastric burning (p = 0.003, 95% CI = 0.10-0.45), and heartburn (p = 0.209, 95% CI = -0.04-0.20).

Conclusions

Based on our findings ginger can be considered as a promising alternative supplementary medicine for FD.

## Introduction

Functional dyspepsia (FD) is among the most common gastrointestinal disorders that affect more than 20% of the population [1]. According to Rome IV criteria, FD is defined as any combination of the following four specific symptoms: postprandial fullness, early satiety, epigastric pain, and epigastric burning symptoms with plenty of interference with the patient’s normal activities and occurring with a frequency of at least three days per week over three months, with an onset of at least six months previously [1].

The prevalence of FD varies worldwide, with a higher occurrence in Western countries (10-40%) [2]. According to a recent study, the percentages of FD symptoms were as follows: postprandial fullness (80%), epigastric distention (80%), early satiety (60-70%), epigastric discomfort and burning (60-70%), nausea (60-40%), and vomiting (80%) [3]. FD is an intermittent condition. According to population surveys, 15% to 20% of individuals experience chronic symptoms over an extended period of time, whereas 50% achieve full symptom relief. There is no evidence linking it to a lower survival rate. Patients with FD have a low quality of life and incur high social and medical expenses. This is in part because conventional FD treatments, which focus mostly on symptom alleviation, are ineffectual [2,4].

The etiology of FD is likely multifactorial; however, the exact cause is not clear. Numerous risk factors can be associated with FD [1]; for instance, enteric infections, being overweight, smoking, antibiotic use, use of non-steroidal anti-inflammatory drugs, and psychological dysfunction are some of the risk factors. It is difficult to manage/treat FD because the primary approach is based on symptom control [5]. Overall, 40% of FD patients worldwide seek medical attention for medication due to severe discomfort and negative consequences on daily activities and productivity [6]. Unfortunately, conventional medications are ineffective in treating FD, and up to 50% of patients turn to alternative therapies such as herbal remedies [7]. As the impact of FD on people’s lives is too great to ignore, it is imperative that scientists investigate and develop new improvements for this condition. Over the previous years, there has been a significant increase in the usage of natural or alternative treatments [8].

Ginger constituents for the management of functional dyspepsia symptoms

Ginger (*Zingiber officinale*) is one of the most consumed herbs and is used both as a spice and dietary supplement worldwide. It has also been used in traditional medicine for its benefit. Chinese and Indians have been using it as a traditional medicine for many years to treat gastrointestinal issues such as indigestion, flatulence, nausea, vomiting, and fever [9]. Studies have indicated that ginger contains a wide variety of chemical constituents of volatile and non-volatile compounds. Some of the stated biological effects are monoterpenes (limonene, citral) and phenolics (gingerols, 6-shogaol, 6-paradol, and zingerone). Limonene, a component of the oil, is used in anticancer and gastroesophageal reflux relief [10], as well as antioxidant, anti-inflammatory, antiviral, and gastroprotective agents [11]. The rhizomes are the most important part, and zingerone, paradols, and shogaols render pungency to ginger [12,13].

Ginger has gained acceptance for its potential to treat various aspects of gastrointestinal symptoms, with in vitro and animal data supporting the antioxidant, anti-inflammatory, anticancer, hypolipidemic, and hypotensive effects [14,15]. Numerous studies have investigated the effectiveness of ginger in treating and improving FD symptoms [16-18]. One randomized, double-blind, placebo-controlled study reported that ginger reduced symptoms of dyspepsia, including epigastric discomfort, compared to placebo. The study involved 70 participants with FD who were randomized to receive either ginger or a placebo for 28 days. The ginger group experienced a significant reduction in symptoms compared to the placebo group [18]. Another study compared the effects of ginger and a proton pump inhibitor (PPI) on dyspepsia symptoms in patients with FD. The study included 60 patients who were randomized to receive either ginger or a PPI for four weeks. Both treatments were effective in reducing symptoms of dyspepsia, including epigastric discomfort, with no significant difference between the two groups [19]. A report from a systematic review of randomized controlled trials (RCTs) found that ginger was more effective than a placebo in reducing symptoms of dyspepsia, including epigastric discomfort. The review included 12 studies involving 1,278 participants [20].

Ginger has also been studied for its effects as an anti-emetic agent on other digestive complaints, including nausea and vomiting [21-27]. A meta-analysis of RCTs found that ginger was effective in reducing nausea and vomiting in various clinical settings, including postoperative nausea and vomiting, chemotherapy-induced nausea and vomiting, and pregnancy-related nausea and vomiting [22]. One randomized, double-blind, placebo-controlled study involving 80 naval cadets found that ginger significantly reduced the severity of motion sickness compared to placebo. The cadets received either ginger or a placebo for four days and were exposed to a motion sickness simulator. The ginger group experienced a significant reduction in the severity of motion sickness compared to the placebo group [23]. A systematic review and meta-analysis of RCTs on the use of ginger for pregnancy-related nausea and vomiting found that ginger was more effective than a placebo in reducing the severity of nausea and vomiting [24]. Even though ginger was found to be effective in most studies in preventing nausea, some clinical studies have been found contradictory [28,29]. It has been indicated that ginger shows no beneficial effects in reducing acute or delayed nausea and vomiting when combined with 5-HT3 receptor antagonists [29].

Several studies have investigated the benefits of ginger supplementation on FD symptoms of bloating and cramps. An RCT published in 2015 found that ginger supplementation significantly reduced FD symptoms, such as upper abdominal cramps and bloating, compared to a placebo. Another randomized controlled trial published in 2016 found that ginger supplementation was associated with a statistically significant reduction in FD symptoms compared to a placebo [30]. A systematic review of six RCTs published in 2018 found that ginger supplementation showed a statistically significant reduction in FD symptoms compared to a placebo. The pooled mean difference in symptom scores was -1.26 (95% CI = -1.89 to -0.62). A meta-analysis of five RCTs published in 2020 found that ginger supplementation was associated with a statistically significant reduction in FD symptoms compared to a placebo. The pooled standard mean difference in symptom scores was -0.65 (95% CI = -1.19 to -0.11). Another human study showed that eating ginger improves digestion by helping the stomach empty faster [31], and a study published in the Journal of Digestive Diseases found that ginger was effective in reducing symptoms of dyspepsia, including stomach pain and bloating [32]. On the other hand, studies have reported that the use of ginger supplementation can affect the improvement of FD [14,18]. The ginger dosage, form, and duration of supplementation were different between studies which might affect the results, and it is also unclear whether ginger is effective for all types of dyspepsia or if it is more effective for certain subtypes. Therefore, further studies are necessary to confirm these findings and understand the ideal amount, form, and duration of ginger supplementation for FD symptoms. Thus, this study aimed to assess the effect of ginger supplementation on the improvement of dyspeptic symptoms in patients with FD.

## Materials and methods

Study population and design

This study was a before-and-after clinical study conducted at the University of Debrecen, Internal Medicine outpatient department among patients with FD. Patient data were collected through a self-completed questionnaire during hospital visits at two study time points, before ginger supplementation and after the fourth week of ginger supplementation. Patients were included based on FD symptoms reported during the initial visit or on the medical database. Patients with peptic ulcer diseases, colon or gastric cancers, pancreatic biliary disease, thyroid disorders, and a positive result for *Helicobacter pylori*; patients who were on pharmacological substances that can affect the digestive system; and pregnant women were excluded.

Intervention

A ready-made product of ginger capsule supplement (Swanson 540 mg ginger root extract, Budapest, Hungary) was used in this study. The enrolled patients were asked to take two capsules (each capsule 540 mg) per day, one 30 minutes before lunch and the other before dinner for four weeks. All patients were asked not to use prokinetic and antisecretory drugs 30 days before the start of the study and during the 28 days of ginger supplementation treatment. During the study period, study participants were encouraged to maintain their daily dietary habits and maintain their normal activities.

Swanson Ginger Root 540 mg Supplement

Swanson Ginger Root 540 mg is a dietary supplement that is designed to support digestive health and promote overall wellness. The following are the specifications of the product: each capsule contains 540 mg of ginger root (*Zingiber officinale*) powder, which is equivalent to 1.35 g of fresh ginger root. The ginger root is standardized to contain a minimum of 4% volatile oils, which are the active compounds responsible for its health benefits. The capsules are made from gelatin, which is derived from bovine sources. The recommended dosage is one capsule taken one to three times daily with water, preferably with a meal. The product is free from artificial colors, flavors, and preservatives, and is also free from common allergens such as wheat, peanuts, eggs, fish, tree nuts, soy, milk, and shellfish.

Swanson Health follows Good Manufacturing Practices (GMPs) to ensure the quality, purity, and potency of their supplements: This information can be found on the Swanson Health website and is standard practice for dietary supplement manufacturers. GMPs are guidelines established by the United States. The Food and Drug Administration ensures that products are consistently manufactured and quality-controlled [33].

Assessment and outcome

Dyspepsia symptoms were asked using the Rome IV questionnaire before and after the supplementation of ginger capsules to assess the effect of ginger on the improvement of FD symptoms. The question about dyspepsia symptoms was graded through the visual analog scale.

Quality assurance

The quality of the study was assured by using a standard questionnaire (Rome IV questionnaire), appropriate selection of participants, translation of questionnaires to the local language (Hungarian), and follow-up during the study period. The enrolled participants were asked and assessed based on the questionnaire if there were changes in FD symptoms after ginger supplementation.

Ethical considerations

The Medical Ethics Committee of Debrecen University approved this research (reference number: DE RKEB/IKEB 5622-2020). Participation was voluntary and all volunteer participants provided written informed consent. All patient data were entirely anonymized, and the study was conducted in accordance with the ethical standards recognized by the Declaration of Helsinki.

Statistical analysis

After coding, SPSS software version 26 (IBM Corp., Armonk, NY) was used for data analysis. Wilcoxon signed-rank test was used to estimate the differences in FD symptoms after ginger supplementation and at the baseline. The difference between the baseline (before supplementation) and after the supplementation was calculated, and the statistical procedure was the repeat t-test. The p-value was determined to test the association between variables with p-values <0.05 considered the cutoff for the statistically significant association.

## Results

Basic characteristics of study subjects

**Figure 1:**
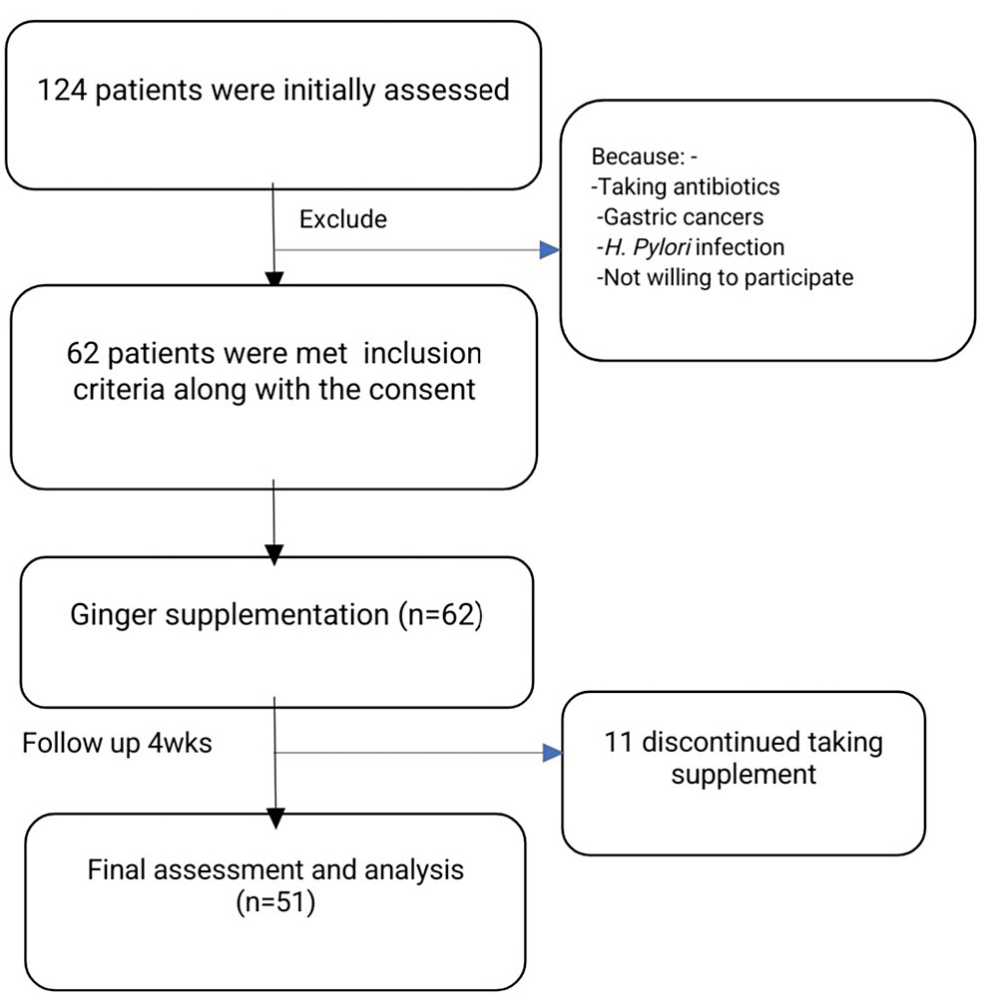
Flow diagram of the study subjects.

In this study of the total 124 initially assessed FD patients, 51 completed the four-week ginger supplementation (Figure 1). The majority of the study participants were females (40/51) which represented 78.4% of the study sample. The basic characteristics of the study subjects are described in Table 1.

**Table 1:** Basic characteristics of study subjects (N = 51). Sex is shown as number (N) and percentage (%) and age and BMI are shown as the average ± SD. BMI = body mass index; SD = standard deviation

Variables	
Sex	N(%)
Male	11 (21.60)
Female	40 (78.40)
	Mean ± SD
Age (years)	49.74 ± 16.38
BMI (kg/m^2^)	25.67 ± 4.90

Frequency distribution of functional dyspepsia symptoms

FD symptoms after ginger supplementation showed improvement in 63.7% of subjects. A moderate improvement was observed with respect to postprandial fullness and early satiety, used to clinically classify subjects with postprandial distress syndrome, which was reduced by about 20% and 30%, respectively, in the scores versus baseline. Epigastric pain and nausea were used to score and clinically classify patients with epigastric pain syndrome, reduced by about 44 and 43%, respectively, in the scores versus baseline. A smaller reduction of approximately 12% was observed for heartburn compared to baseline (Table 2).

**Table 2:** Frequency distributions in FD symptoms at baseline and after 28 days of ginger supplementation (N = 51). FD = functional dyspepsia

Symptoms	Baseline	28 days of ginger supplementation
Postprandial fullness	36 (70.6%)	29 (56.9%)
Early satiety	40 (78.4%)	28 (54.5%)
Epigastric pain	34 (66.7%)	19 (37.3%)
Nausea	44 (86.3%)	25 (49%)
Heartburn	33 (64.7%)	29 (56.9%)

## Discussion

The results from this study indicated that ginger supplementation was associated with a statistically significant reduction in most FD symptoms. Postprandial fullness (p = 0.033, 95% CI = 0.01-0.26), early satiety (p = 0.001, 95% CI = 0.10-0.37), epigastric pain (p = 0.000, 95% CI = 0.16-0.42), epigastric burning (p = 0.003, 95% CI = 0.10-0.45, and heartburn (p = 0.209, 95% CI = -0.04-0.20) showed highly statistically significant findings while for heartburn smaller decreases were seen in comparison to baseline (Table 3), which was consistent with previously conducted studies [34,35]. A randomized, double-blind, placebo-controlled study found that ginger supplementation reduced symptoms of dyspepsia, such as belching, bloating, and fullness [34]. Additionally, a randomized, double-blind, placebo-controlled study published in the European Journal of Gastroenterology and Hematology found that ginger supplementation reduced symptoms of FD, including postprandial fullness and early satiation [35]. According to a study done on FD patients, eating 1.2 g of ginger powder significantly enhanced antral contractions and stomach emptying compared to taking a placebo [18]. Ginger may accelerate gastrointestinal peristalsis and shorten the time it takes for food to move through the digestive tract via modulating intestinal 5-hydroxytryptamine (5-HT; serotonin) receptors [36,37].

**Table 3:** Comparison of symptoms of functional dyspepsia (average ± standard deviation) at baseline and after 28 days of ginger supplementation (N = 51). * = statistically significant evidences (p < 0.05).

FD symptoms	Baseline	28 days of ginger supplementation	P-value (95% CI)
Postprandial fullness	0.70 (0.46)	0.57 (0.05)	0.033* (0.01-0.26)
Early satiety	0.78 (0.42)	0.55 (0.50)	0.001* (0.10-0.37)
Epigastric pain	0.67 (0.48)	0.37 (0.49)	0.000* (0.16-0.42)
Epigastric burning	0.59 (0.50)	0.31 (0.47)	0.003* (0.10-0.45)
Nausea	0.86 (0.35)	0.49 (0.50)	0.000* (0.21-0.53)
Heartburn	0.65 (0.48)	0.57 (0.50)	0.209 (-0.04-0.20)

As the causes of FD are multifaceted [6], various pathophysiological mechanisms may account for the etiology of FD, such as impaired meal-induced relaxation of the proximal stomach, visceral hypersensitivity to distension, gastric motor abnormalities, and disturbed central nervous function [38]. This multifactorial and poorly defined pathogenesis has hampered efforts to develop effective treatments in most FD cases [39], and its optimal clinical management remains a subject of considerable debate [40]. The typical method for treating patients with suspected FD considers a test-and-try approach to reduce symptoms as a potential starting strategy. The first possibilities in such a strategy are PPIs and/or prokinetics [41,42]. Motivated FD sufferers may take into account herbal remedies as alternatives. Recently, a number of supplements with a focus on ginger extracts have been suggested to physicians and gastrointestinal experts. The nutritional supplement industry offers these extracts in specific quantities made from herbal or spice ingredients [43].

Most animal studies have shown that ginger root extract increases gastric ulceration and gastrointestinal transit [44], which may contribute to our results. Ginger has been suggested to promote gastric emptying and improve gastrointestinal motility [45]. A clinical study showed the effects of ginger on indigestion in 12 healthy volunteers. The study found that ginger significantly accelerated gastric emptying compared to a placebo [46]. In another study, 24 patients with FD were randomized to receive either ginger or a placebo for 14 days. The study found that ginger significantly improved gastric emptying compared to a placebo [19]. However, a study involving 14 healthy adults found no significant effect of ginger on gastric emptying. The study used a randomized, double-blind, crossover design and found that ginger did not significantly affect gastric emptying than a placebo [47].

In this study, we observed an association between ginger supplementation and nausea. Vomiting, nausea, and hypomotility involve a transient dysfunction of the complex integrated web of cholinergic M3 and serotonergic 5-HT3/HT4 receptors. The main constituents of ginger root extracts such as gingerol-6, 8, 10, and 6-school have been reported in many experimental models to modulate these receptors. On the other hand, 5-HT4 receptors, which also play a role in gastroduodenal motility, do not seem to be involved in the effects of these compounds [23,48]. This discrepancy may stem from variations in supplementation amount, form, and duration between studies, which could have an impact on findings. Overall, the data indicate that ginger supplementation may be effective in improving dyspepsia symptoms. However, more well-designed studies are needed to approve these findings and determine the appropriate dosage and duration of supplementation.

Limitations of the study

Our study was a single-group study and was not placebo-controlled, where the findings would carry more weight. Our study was limited by its small sample size of only 51 patients.

## Conclusions

Based on the findings of this study, ginger and its constituents can be considered alternative supplementary medicine for FD. However, due to the small number of patients and the study design, more well-designed studies are needed to approve these findings.
